# The Psychical Effects of Ether Inhalation

**Published:** 1854-12

**Authors:** Moreton Stillé


### *The Psychical effects of Ether Inhalation.*

By Moreton Stillé, M. D.

A very short time after the first announcement of the pain-destroying properties of ether and chloroform, the facility with which it was supposed that these agents might be used for criminal purposes, was strongly urged as an objection to their employment. The rapid and universal adoption of these beneficent means for the annulling of pain, caused these unfavorable auguries to be forgotten; and, fortunately, experience has shown that the objection, although not indeed imaginary, was of but slight importance. It is, indeed, true that persons were said to have been waylaid in the streets or surprised in their houses, and being forcibly compelled to inhale the vapor of chloroform from an unseen hand, were plundered of money and other valuables. But the reality of any such occurrences is more than questionable; the instinctive struggles to resist the inhalation would inevitably defeat the object of the criminal, and expose him to the risk of being recognized. Dr. Snow, of London, whose thorough familiarity with the phenomena attending the inhalation of chloroform is well known, ridicules these reports, and suggests that they may have had their origin in the misadventures of those whose potations had been too deep for their good. A statute was, however, enacted by Parliament, (14 and 15 Vict. ch. 19, s. 3,) making the use of ether and chloroform a felony, where employed for the purpose of producing insensibility, with intent to commit rape or other felonies. The only instance, as far as we know, of alleged rape under these circumstances, is one which occurred in Paris, but the full details of which are not to be found in any of the journals, the trial having been conducted with closed doors. Two young girls brought a charge of rape against a dentist, alleging that the act had been perpetrated while they were under the influence of ether, and that they were incapable of resisting it. As far as we can discover from the imperfect report of the charge of the presiding judge, no other testimony was offered to the fact but that of the young women; the accused was condemned to six years of hard labor, and damages to the amount of 1500 francs. "He retired," it is said, "protesting his innocence."—(Abeille Médicale, 1847, p. 331.)

The trial which has been recently concluded in this city, for a similar offence alleged to have been committed by a dentist upon one of his patients, has awakened a warm interest in the community. It is not our purpose to make any direct allusion to this trial; nor to offer an opinion upon the testimony brought forward to sustain the accusation. Our remarks, although suggested by this painful event, have a wider scope, and are intended to bear upon the general medico-legal question of the value of evidence given by an etherized patient of what has occurred during the period of etherization. It is certainly of the highest importance, that a question involving both the honor of the woman and the liberty, good name and fortune of the man accused of outraging it, should receive all the light which the manifold researches of physiologists and medical practitioners are capable of throwing upon it. If an accusation of rape is one "easily made and hard to be refuted," this is particularly the case under the circumstances referred to, and it may at any time be successfully made against the purest and best men in the community.

There is a striking analogy between the effects of ether and those of alcohol; the chief difference between them being in the more rapid and complete insensibility produced by the former, and in the more evanescent character of the intoxication. There is a period of exitement, of stupor, and of recovery, and the phenomena observed in different individuals vary according to their temperament and habits. In general, the stage of excitement in etherized patients is short, and verges rapidly into that of unconsciousness and insensibility to pain. The vapors of ether seem literally to ascend and diffuse themselves through the brain and to permeate every portion of the body; the patient has a sense of fulness and warmth, the whole body feels lighter and seems to spurn the earth; the sense of hearing becomes confused, the sight dim and the touch benumbed. External objects lose themselves in a confused mist, which appears to swell their proportions and contort their shape, the muscles become relaxed, and the patient sinks lethargic and unconscious into a profound sleep.

During the transition into the stage of entire insensibility, he responds to external impressions only in an automatic manner; the most painful incisions, if felt at all, seem to him like the marking out of lines upon the skin, and the extraction of deep seated tumors like the crackling of hair between the fingers. All his movements are instinctive, an expression of suffering is often depicted upon the face, the hands are raised against the operator as he attempts to draw a tooth, and when spoken to, he answers in a vague and dreamy manner. The recovery from this condition, or from a more advanced stage, is apparently sudden, but as in the waking from profound natural sleep, the perceptions are for a few moments confused, even while the person thinks himself fully awake and appears to be so.

Dr. Forbes has well described the *psychical* state under the influence of ether. "Generally speaking," he says, "the sense of external impressions becomes at first confused, then dull, then false, with optical spectra or auditory illusions, general mental confusion and then a state of dreaming or utter oblivion. In the majority of cases, the mind is busy in dreaming, the dreams being generally of an active kind, often agreeable, sometimes the reverse, occasionally most singular; and, frequently, a great deal is transacted in the few short moments of this singular trance. Many of the patients who have undergone the most dreadful operations, such as amputation of one or both thighs or arms, extraction of the stone, excision of bones, extirpation of the mamma, have readily detailed to us, and most with wondering thankfulness, the dreams with which and with which alone, they were occupied during the operations. The character of the dreams seemed to be influenced, as in ordinary cases, by various causes immediate or remote, present or past, relating to events or flowing from temperament." * * * * * "A good many seemed to fancy themselves on the railway, amid its whirl and noise and smoke; some young men were hunting, others riding on coaches, the boys were happy at their sports, in the open fields or the filthy lane; the worn Londoner was in his old haunts carousing with his fellows; and our merry friend, Paddy, of the London Hospital, was again at his fair, wielding his shilela in defence of his friends. Others of milder mood, and especially some of the women patients from the country, felt themselves suddenly transported from the great city and the crowded hospital-ward, to their old quiet home in the distant village, happy once more with their mothers and brothers and sisters. As with the dying gladiator of the poet, the thoughts of these poor people—

"Were with their heart, and that was far away."

Some seemed transported to a less definite, but still happy region, which they vaguely indicated by saying they were in heaven; while others had still odder and warmer visions which need not be particularized."—(Brit. & For. Med. Chir. Rev. April, 1843.) It is with this psychical condition that we have now chiefly to do.

What then is the influence of the inhalation of ether upon the *perceptions?* It undoubtedly cuts off, more or less quickly, the life of relation, and severs us from the external world. The lapse into unconsciousness is gradual but rapid, and does not admit of division into distinct intervals. The sensation of pain is often lost before outward consciousness has become totally obscured. Indeed, instances are related in which the patient has himself looked on as a calm spectator of the painless mutilation of his body. A patient of Prof. Pitha, being put under the influence of chloroform, at once fancied himself in his beloved Italy, and gave full vent to his expressions of delight; he raised himself up during the operation for the liberation of a hernia, and watched it with great interest, answering to the question whether he felt any pain, "*Si io sento l'incisione, ma non sento dolori.*" (Prager Viertel jahrschrift, 1848. 3 Bd.) Such cases are rare, and it is important that we should not be misled by this apparent outward consciousness. In the instance just cited, the perception was by no means unperverted; since, although the patient replied correctly when questioned, he imagined himself in a distant country. During an extremely painful operation performed by Velpeau, upon a young girl, she raised herself into a sitting position, as if to observe it. She said afterwards that she supposed herself seated at a dinner table.—(Rev. Med. 1847.) In the greater number of cases, however, the perceptions are greatly perverted, illusions being sometimes suggested by the scene actually passing, and at others arising without being prompted by external perceptons. Some cases illustrating this fact, we quote from the interesting work of Dr. Flagg.—(Ether and Chloroform, &c., by J. F. B. Flagg, M. D., Surgeon Dentist, &c. Philadelphia, Lindsay & Blakiston, 1851.)

After an operation performed upon the forehead of Mr. T——, a dentist of this city, he said that although his eyes were shut, he saw every cut of the knife. "He saw the shape of the wound upon the forehead; and what was better than all, this cutting appeared to him to be done upon somebody else." A lady dreamed that she was at Cape May, and was going into the surf, and that while in the water, she was attacked by a shark, which held her fast, but without pain, until the company present extracted his teeth and liberated her. A little girl, the extraction of whose tooth made a report like the drawing of a cork, sprang out of the chair "crouched upon the floor, and looked up anxiously at me and inquired if anybody was killed." She supposed that shs was travelling upon a locomotive engine, which had been blown up and thrown her into the air. A boy fancied himself in a cotton mill; an Irishwoman dreamed that she had been home, and seen her friends engaged in spinning, and others dreamed that they were in railway cars or shipwrecked; the dream in some cases being suggested intentionally by the dentist, or being due to accidental noises. A countless number of cases might be adduced to show that patients under the influence of ether, have been completely ignorant of all that has passed around them while in this condition; and have been surprised to find upon their recovery that they have undergone the most severe surgical operations. But this fact is too familiar to need illustration. It is only important to observe that during this state of utter oblivion, the mind is often busily engaged upon its own inward perceptions, which may or may not be pertinent to the actual position of the patient. These perceptions shape themselves into dreams entirely similar to those of natural sleep, being grotesque and improbable, cheerful or painful, according to the temperament, occupation and habitual modes of thought of the individual.

One of the most extraordinary effects of the inhalation of ether, is its effects upon the *emotions*. Thus some persons are seized with the most irrepressible mirth, while others seem to sink under the weight of despondency. Women are especially liable to these effects. Hysterical paroxysms are by no means a rare accompaniment of ether inhalation. In others, the erotic propensities are strangely excited. Siebold relates the case of a woman whom he rendered insensible by ether; upon regaining her consciousness, she appeared to be in a highly excited state, and was loud in her praises of the delightful condition in which she had been, her eyes sparkled and a certain erotic excitation was very observable.—(Uber die Anwendung der Schwefel —Æther—Dämpfe in der Geburtshülfe, Göttingen, 1847.) Pitha observed excitement of the sexual feelings in two cases, one of a woman and the other of a man upon whom he operated. (Prager viertel jahrschrift, 1847, Bd. 3.) "In one of the cases observed by M. Dubois, the woman drew an attendant towards her to kiss, as she was lapsing into insensibility, and this woman afterwards confessed to dreaming of coitus with her husband while she lay etherized. In ungravid women, rendered insensible for the performance of surgical operations, erotic gesticulations have occasionally been observed, and in one case, in which enlarged nymphæ were removed, the woman went unconsciously through the movements attendant on the sexual orgasm in the presence of numerous bystanders." (A Lecture on the utility and safety of the inhalation of Ether in Obstetric Practice, by W. Tyler Smith, M. B., Lancet, Mar. 27, 1841,) also in (Bulletin de l'Acadamie, vol. 12, p. 406.) We doubt not that other cases might be brought forward to illustrate this fact, but the paucity of published reports of such a nature, will be readily attributed to the natural unwillingness of patients to disclose painful illusions of this kind, and of physicians to make them known. In further illustration of the disordered condition of the mind under the influence of ether, the following case may be cited. A female rendered insensible by ether, after some unintelligible phrases, related somemost circumstantial details of her private life. This involuntary confidence, which might have been followed by serious consequences, had it taken place anywhere but in an hospital, was discovered afterwards to have been perfectly true.—(Ann. Médico-psycholog. vol. xii. 376.)

In the above observations, it may very plainly be seen that the *will* no longer exercises its control over the mental operations. The thoughts run headlong upon their accustomed track, or in any direction in which they may have been impelled by fortuitous impressions, made upon the nerves of general or special sensation. There is no power to restrain them, and while the dream is a pleasant one, no desire to do so. Often, however, the illusions are painful or disagreeable, and in such cases, the individual may make an effort to escape from or to repel them. Movements under these circumstances, therefore, imply an exercise of the will. This resistance is almost always to illusions proceeding from external impressions. We have already referred to the frequent occurrence of instinctive struggles against the hand of the operator, while the impression, as afterwards related, has been upon the mind of the patient, that he was playing a part in some very different scene. Thus the little girl, whose case is before referred to, and who fancied, when her tooth was drawn, that she was blown from a locomotive, *sprang* from her chair upon the floor, while still unconscious.

Another young lady, mentioned by Dr. Flagg, when the forceps was placed upon the tooth, cried out, "stop pulling! stop pulling!" The tooth was nevertheless extracted. "She *rose* from the chair in much excitement, and would have fallen to the floor, but I caught and sustained her for a moment, when the ether instantly passed off." This young lady dreamed that she was in danger of shipwreck, and seeing the rocks and breakers ahead cried out to the man at the wheel with all her strength to "stop pulling." In another instance a lady while under the influence of ether resisted the attempt to extract her tooth. She *got up* from the chair, seeming much offended, and took her seat in another part of the room. When the effect of the ether passed off, which was in about a minute, she was much astonished at finding herself so remote from the position she occupied when she fell asleep.—(Flagg, p. 102.)

The following singular instance may be appropriate in this place. A young man having been sufficiently etherized, the dentist prepared to extract a tooth. In a moment he dashed the instrument from his mouth, *left the chair*, and striding about the room demanded what they meant to do with him. In a few moments the effect of the ether passed off. Being again put under its influence the same scene was enacted with even greater violence, and he endeavored to jump out of the window. When he regained his memory he related that he imagined himself surrounded by a great number of enemies, one of whom endeavored to drive a nail into his mouth, and being unable to struggle with them he had sought safety in flight.—(Union Med., Sept. 1847.)

M. Gerdy in trying the effect of ether upon himself, with the object of observing closely its successive phenomena, found that with the exception of the vibratory and benumbed sensation which rendered the sense of touch and of pain obtuse, and the noise in the ears which dulled the sense of hearing, his intelligence was clear, his attention active, and his *will* so firm that he willed to walk and he did walk, in order to observe the effect upon his locomotion. He found that his step was only less sure than usual, and was similar to the progression of an intoxicated person.—(Bulletin de l'Acadamie, vol. 12, p. 304.)

We have cited these examples, out of many of a similar nature, for the purpose of showing that the power of the will over muscular movement is not entirely abolished in etherization. It is true that the muscles are speedily relaxed, but they are not paralysed. The patient may exercise his will or he may not; if he does, it is to escape from danger, real or imaginary, but which has always to him the form of reality. If he does not make any movement, the fact is due either to the pleasurable or trivial character of his mental perceptions, or to the temporary but complete unconsciousness and insensibility in which he is plunged. That advanced stage of etherization in which perfect narcotism is produced, is, in reference to the present question, of considerable importance; for if the power of resistance is then lost, so also is the consciousness of a real motive for it. To be more explicit, if an outrage be perpetrated upon a woman lying wholly helpless and unconscious, she cannot be aware of the liberties which are being taken with her person, and will not, therefore, make any opposition to them. She cannot, moreover, afterwards describe with elaborate detail the manner and particulars of the assault, and yet have been incapable of withdrawing from or repelling it. If her muscles and her voice have been paralysed, so also has her outward conciousness.

The recollection of what has passed during this stage of etherization is wholly confined to the inward mental perceptions, to the dreams which have all the vividness of real occurrences. In the language of Dr. Forbes, "the old story of the magician in the Arabian tales seems more than realized, the ether being like the tub of water, one moment's dip of the head into which produced a life-long vision in the dreamer's mind." It is possible that these dreams may be so vividly impressed upon the mind that they may have afterwards to the patient all the force of real occurrences, and that he may refuse to believe that they have been merely the disordered perceptions of his own brain. In general, these dreams being of a trivial or of a pleasing character, it is not surprising that the patient should acquiesce in the belief of their unreal nature, but the case is very readily conceivable in which the hallucination may have been so distinct and at the same time of so repulsive a character as to leave an indelible impression upon the mind and a conviction of its reality. Authentic published evidence of this fact is indeed wanting, and we purposely forbear, for reasons which cannot fail to be apparent to our readers, to refer to that which is said to have been offered in the recent trial, as well as to that which we possess from private sources.

The following cautious remarks of M. Bayard are not without significance: "If," he says, "in some cases, individuals have rendered an exact report of what has passed around them, or of the liberties which have been taken with them while under the influence of ether and chloroform, it must not be forgotten that very frequently they have dreams, hallucinations, and illusions which they relate with a conviction of their actual reality. Experts should therefore receive with extreme circumspection declarations made before them under these circumstances, and both in their written reports and verbal depositions should endeavor to enlighten magistrate and jury upon the relative value and credibility of such revelations."—(Appréciation médico legale de l'action de l'ether et du chloroforme. Ann. d'Hygiene, vol. 42, p. 201.) It appears to us, from what has now been stated that the following positions may be assumed as correct.

1st. That the consciousness or perception of external objects and impressions is impaired in the early and lost in the final stage of etherization.

2d. That during the time the mind remains susceptible to external impressions at all, these reach it in a feeble or perverted manner.

3d. That the emotions, and especially those of an erotic character are excited by the inhalation of ether.

4th. That voluntary muscular movement is not paralysed until the state of perfect narcotism is produced, at which time, however, all outward consciousness is extinct.

5th. That the memory of what has passed during the state of etherization is either of events wholly unreal, or of real occurrences perverted from their actual nature.

6th. That there is reason to believe that the impressions left by the dreams occasioned by ether, may remain permanently fixed in the memory with all the vividness of real events.

The practical inferences from these positions are too plain to need explanation; we commend them, therefore, to the reader's meditation.

